# Classifying leukemia types with chromatin conformation data

**DOI:** 10.1186/gb-2014-15-4-r60

**Published:** 2014-04-30

**Authors:** Mathieu Rousseau, Maria A Ferraiuolo, Jennifer L Crutchley, Xue Qing David Wang, Hisashi Miura, Mathieu Blanchette, Josée Dostie

**Affiliations:** 1 Department of Biochemistry and Goodman Cancer Research Center, McGill University, Montréal, Québec H3G 1Y6, Canada; 2 McGill Centre for Bioinformatics and School of Computer Science, McGill University, Montréal, Québec H3G 0B1, Canada

## Abstract

**Background:**

Although genetic or epigenetic alterations have been shown to affect the three-dimensional organization of genomes, the utility of chromatin conformation in the classification of human disease has never been addressed.

**Results:**

Here, we explore whether chromatin conformation can be used to classify human leukemia. We map the conformation of the HOXA gene cluster in a panel of cell lines with 5C chromosome conformation capture technology, and use the data to train and test a support vector machine classifier named 3D-SP. We show that 3D-SP is able to accurately distinguish leukemias expressing MLL-fusion proteins from those expressing only wild-type MLL, and that it can also classify leukemia subtypes according to MLL fusion partner, based solely on 5C data.

**Conclusions:**

Our study provides the first proof-of-principle demonstration that chromatin conformation contains the information value necessary for classification of leukemia subtypes.

## Background

The organization of the human genome in the nucleus is non-random and important for proper gene expression [1-4]. For instance, chromosomes are known to occupy distinct regions called ‘chromosome territories’ that are anchored to the lamina. Gene-rich chromosomes tend to localize at the center of the nucleus and gene-poor near the periphery [5-9]. The co-localization of co-regulated genes into transcription factories is another key feature of genome organization that is thought to coordinate and/or potentiate transcriptional responses [5-9]. Chromatin architecture can also control transcription by promoting or restricting physical proximity between distal control DNA elements. As such, long-range contacts can correlate with either activation or repression of transcription [10-12]. For example, transcription activation at the β-globin locus associates with physical contacts between the locus control region (LCR) and actively transcribed genes [13]. Conversely, insulator (CTCF)-mediated loops differentially compartmentalize inactive genes away from enhancers at the apolipoprotein locus [14]. The relationship between architecture and expression is also well illustrated by the inactivation of one X chromosome in the nuclei of female mammalian cells [15], where epigenetic silencing leads to a condensed chromatin structure with silent genes at the core and expressed ones looped out [16].

Given the relationship between genome architecture and activity, it is not surprising that human disease can sometimes be attributed to defects in genome organization. Compelling evidence for the role of three-dimensional (3D) chromatin organization in human disease comes from studies on laminopathies like the Emery-Dreifuss muscular dystrophy. It was shown that this disease could originate from mutations in a lamin protein that specifically causes abnormal retention and silencing of muscle-specific genes at the nuclear envelope [17]. The importance of spatial genome organization in human disease is equally well demonstrated in cancers where single nucleotide polymorphisms (SNPs) were found to create novel enhancers acting long-range to activate distal genes through DNA looping [18-20].

Overall, genome architecture is guided by chromatin interactions with nuclear landmarks like the lamina, and by intra- and inter-chromosomal contacts mediated by chromatin-binding proteins. In addition to transcription factors that form DNA loops between enhancers and promoters, proteins like CTCF, SATB1, and the Cohesin complex are thought to be master regulators of genome organization. Protein complexes such as those containing Polycomb group proteins or the mixed lineage leukemia (MLL) protein might be equally important in shaping the human genome [21-23]. MLL is an H3K4 methyltransferase that is present in COMPASS-like (complex of proteins associated with Set1) complexes [24]. These multi-subunit complexes are very large, and activate transcription partly by methylating H3 on Lys 4. COMPASS-like complexes control the expression of many genes with pivotal roles in development and differentiation including homeobox family members like the *HOX* genes.

The MLL gene is a common target in non-random chromosomal translocations associated with both acute lymphoblastic (ALL) and acute myeloid (AML) leukemia, with over 50 different translocation partners identified so far [25-27]. These translocations result in the production of gain-of-function chimeras composed of an amino-terminus MLL lacking the SET domain fused in frame with another protein coding gene. In all cases, the resulting MLL fusion oncoprotein acts as a strong transcriptional activator that disrupts the normal hematopoietic differentiation program by inducing the aberrant expression of key regulators including *HOX* family members [28]. In fact, dysregulation of *HOX* genes was reported to be a dominant mechanism of leukemic transformation by MLL fusions [29].

The mechanisms by which different MLL fusions activate transcription or lead to either AML or ALL are poorly understood. However, the observation that many translocation partners are elongation factors that co-exist in a super elongation complex (SEC) with the fusion proteins suggests that they can activate transcription at the elongation step [30]. Interestingly, many fusion partners of MLL bind each other to form transcription foci visible by microscopy. Since the expression of MLL chimeras can alter their localization and activity, these findings suggest that MLL fusions might alter chromatin organization [22,31,32]. Also, given that the epigenetic state of chromatin and its conformation are thought to mutually affect each other in self-enforcing structure-function feedback loops [33], it seems likely that MLL fusions additionally alter chromatin organization by modifying its epigenomic profile.

In infants, MLL is translocated in over 50% of acute leukemias [34,35], whereas translocations in adults are most often seen in patients having undergone chemotherapy [36]. In general, the prognosis of AML patients expressing MLL fusions is poor [37], and new classification methods could help identify optimal treatment courses. We previously reported that terminal differentiation of the AML THP-1 cell line into macrophages is accompanied by transcriptional repression and spatial remodeling of the *HOXA* gene cluster [38]. From these results, we wondered whether chromatin architecture, which in essence reflects genome activity, could be used to classify leukemia types. Here we present a study that provides a proof of concept that chromatin conformation can be used to classify leukemias. We found that when the highly relevant *HOXA* region is considered, chromatin architecture has the information value to distinguish between leukemia types and subtypes.

## Results and discussion

### The *HOXA* gene cluster as a test locus

**Figure 1:**
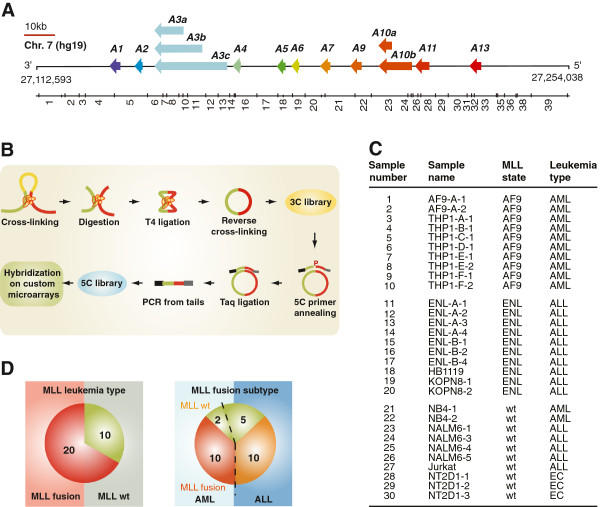
**Experimental design used to generate the training set for 3D-SP. (A)** Linear schematic representation of the human *HOXA* cluster region characterized in this study. Genes are illustrated as left-facing arrows to indicate transcription direction and highlight the 3’ to 5’ end orientation of the cluster. The 11 paralogue groups are color-coded and identified above each gene. *Bgl*II restriction fragments of the *HOXA* region characterized here are shown below and identified by numbers from left to right. **(B)**  Diagram of the 5C technology. 5C quantitatively measures chromatin contacts using primers that are complementary to predicted junctions in 3C libraries. Annealed 5C primers are ligated with Taq DNA ligase and products are amplified by PCR using oligos recognizing the universal tails of 5C primers. In this study, amplification was done using a fluorescently labeled reverse PCR primer and amplified 5C libraries were hybridized onto custom microarrays. **(C)** Leukemia cell panel used to train 3D-SP. Cell lines are organized by leukemia type and MLL status. AF9; AF9 gene (ALL1-fused gene from chromosome 9), ENL; ENL gene (eleven-nineteen-leukemia), wt; wild type, AML; acute myeloid leukemia, ALL; acute lymphoblastic leukemia, EC; embryonic carcinoma. **(D)** Distribution of leukemia cell samples used to train 3D-SP. Left pie chart indicates the distribution of leukemias expressing either MLL fusions or the *wt* protein (Leukemia types). Right pie chart shows the distribution of MLL types (MLL fusion types).

The *HOX* clusters encode transcription factors that are important for embryonic development and hematopoietic lineage regulation [39,40]. Aberrant *HOX* expression is found in various types of human cancers including lung cancer [41], breast cancer [42], melanoma [43], and leukemia [44]. *HOXA9* and *10* for instance are oncogenes overexpressed in various leukemia types and are direct targets of MLL fusion oncoproteins [45-47]. In mammals, there are 39 *HOX* genes organized into 13 paralogue groups and divided into four clusters named *A*, *B*, *C*, and *D* located on different chromosomes [48,49]. The human *HOXA* cluster spans over 100 kbp on chromosome 7 and encodes 11 transcription factors (Figure 1A). To determine whether chromatin architecture can be used to classify disease, we mapped the organization of a region containing the *HOXA* cluster with the chromosome conformation capture carbon copy (5C) technology (Figure 1B). The 5C method is a member of the so-called ‘3C technologies’ used to measure genome organization *in vivo* at high-resolution [50,51]. 5C captures chromatin conformation by converting chemically cross-linked chromatin segments into unique ligation products, which are then detected high-throughput using a modified version of ligation mediated amplification (LMA).

**Figure 2:**
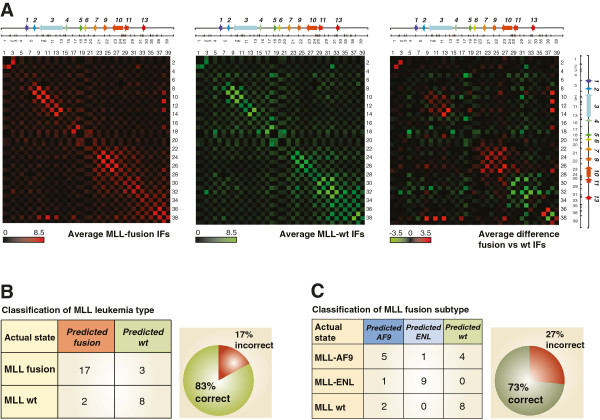
**The *HOXA *cluster conformation can be used to classify leukemia cell samples. (A)** Averaged 5C interaction frequencies from MLL-fusion leukemia cell samples (*left*), cells encoding only the *wt* MLL protein (middle), and difference between the two MLL leukemia types (right). The data from the left panel are the average of the 5C datasets presented in Additional file 4: Figure S2A, and the middle panel contains the averaged data from Additional file 4: Figure S2B. Normalized pair-wise interaction frequencies are color-coded according to the scale shown on the bottom left of each heatmap. Numbers above and on the right of each heatmap identify *Bgl*II restriction fragments corresponding to the restriction pattern shown below the *HOXA* diagrams. Intersecting column and row numbers identify DNA contacts. **(B)** Classification results of the 3D-SP trained to distinguish between samples expressing MLL fusions and the *wt* MLL protein. **(C)** Classification results of the 3D-SP trained to distinguish between samples expressing either MLL-AF9 or MLL-ENL. For **B** and **C**, the leukemia training set shown in Figure 1C was used to train 3D-SP. Results shown are from a leave-one-out cross-validation of the 3D-SPs. The pie chart on the right of each table shows the overall accuracy of the corresponding 3D-SP. Matthews Correlation Coefficient (MCC) = 0.64.

Using an experimental design previously described [38] we measured chromatin contacts throughout the *HOXA* cluster region in a panel of leukemia cell lines (Figure 1C). This panel, which is detailed in Additional file 1: Supplementary Materials and methods, includes 20 samples expressing MLL fusions and 10 with only the *wt* protein (Figure 1D, left). The panel featured AML and ALL caused by a fusion between MLL and the AF9 gene (MLL-AF9; AML), MLL and the ENL gene (MLL-ENL; ALL), or expressing the wild-type (*wt*) protein (Figure 1D, *right*). We also included three embryonic carcinomas (EC) samples in this training set to increase diversity. These are known to encode only the *wt* MLL protein and express no *HOXA* genes ([52] and Additional file 2: Table S1). The normalized 5C data from these samples were derived as detailed in Additional file 1: Supplementary Materials and methods and shown in Additional file 3: Figure S1. The 5C datasets are presented in heatmap form in Additional file 4: Figures S2, and show a very high degree of variability between all samples, regardless of whether they express MLL fusions, the *wt* protein are AML or ALL. When comparing the average *HOXA* interaction frequencies (IFs) in leukemia samples expressing MLL fusions to those encoding only *wt* MLL, we could find marked differences in contact frequencies between neighbors (heatmap diagonal) and between distal fragments interacting long-range (Figure 2A). For example, we observed higher local contacts around the *HOXA3* gene in MLL fusion samples, and more long-range interactions between the 5’ end, the middle and the 3’ end of the cluster in samples where only the *wt* MLL protein is expressed (Figure 2A, right). These results indicate that the *HOXA* chromatin conformation in leukemia cell lines expressing MLL fusions and MLL *wt* might differ sufficiently to be used for classification.

### Development, training, and performance of 3D-SP

Although significant differences could be observed between averaged IFs, this type of ‘direct’ measurement does not reliably identify contacts that most consistently describe a particular leukemia type and that could be used for classification. Indeed, average or greater IFs in a given sample set might simply originate from the presence of a few samples where these contacts are high. To more robustly distinguish between leukemia types, we developed a support vector machine (SVM; [53]) classifier called ‘3D-SP’ (3-Dimensional DNA Disease-Signature Predictor), which uses the complete IF data from a 5C experiment as basis for classification. We opted for an SVM since they were previously shown to yield good accuracy classifiers even for high-dimensional data [54].

3D-SP was evaluated using leave-one-out cross-validation on the set *S* of 30 samples shown in Figure 1C. Specifically, for each sample *s* in *S*, a classifier was trained on the 29 remaining samples (*S - {s}*) and then used to predict the class of *s*. The result of this cross-validation procedure is then reported as one entry in the confusion matrix shown in Figure 2B. This ensures that no predictor was trained using the sample on which it is asked to make a prediction. Using this approach, we found that leukemia samples expressing either MLL fusions or the *wt* protein could be classified with 83% accuracy by 3D-SP (Figure 2B). Training 3D-SP to recognize features specific to MLL fusion subtypes also yielded good classification results by leave-one-out cross-validation albeit with a lower accuracy of 73% (Figure 2C). These results demonstrate that the *HOXA* cluster organization can be used to classify different leukemia types.

### Identifying highly predictive chromatin contacts

**Figure 3:**
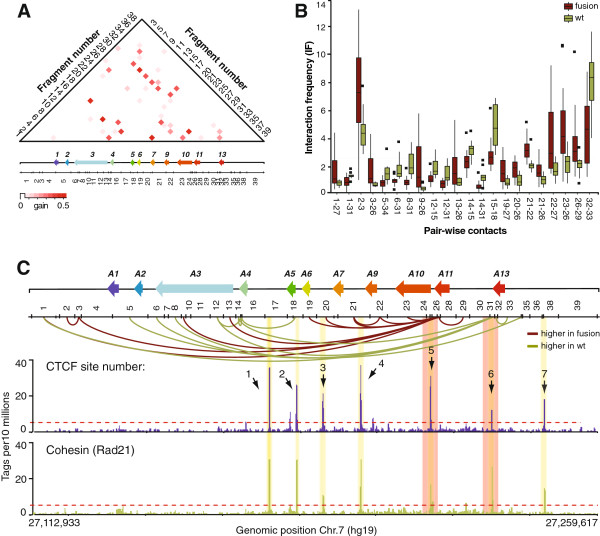
**Distinct contact patterns identify MLL fusion from *wt *MLL leukemias. (A)** Heatmap representation of the information gain scores of contacts throughout the *HOXA* cluster. The values are color-coded according to the scale shown at the bottom of the heatmap. Numbers on the left and right of heatmap identify *Bgl*II restriction fragments corresponding to the restriction pattern shown below the *HOXA* diagram. Intersecting column and row numbers identify DNA contacts. **(B)** Averaged interaction frequencies of 22 contacts with high information gain scores (IF values between MLL fusions and *wt* MLL are statistically different, *P* <0.01). Interaction frequencies in MLL fusion datasets are the averaged values from Additional file 4: Figure S2A, and *wt* MLL values are from Additional file 4: Figure S2B. Error bars represent the standard error of the mean. **(C)** Distribution of informative contacts for classification, and binding of CTCF and cohesin in THP-1 cells along the human *HOXA* cluster region examined. The y-axis shows the number of CTCF ‘Tags per 10 millions’ obtained by ChIP-seq after normalization against input, across the region characterized (x-axis). CTCF peaks are numbered from left to right (CTCF1 to 7). Regions forming contacts with high predictive power are highlighted in orange.

We next wondered which *HOXA* contacts showed the greatest difference between classes and conferred the largest amount of predictive power in the classification of leukemias expressing either MLL fusions or the *wt* protein. By measuring the information gain score of each pair-wise interaction, we found that over 20 different contacts contributed information that enhanced the classification performance (Figure 3A; Student t-test *P* value <0.01). The information gain estimates the reduction of entropy in the classification achieved by each contact, and can therefore be used to identify discriminatory interactions. As expected, there were much fewer contacts than those displaying large averaged IFs differences (compare Figures 2A and 3A). For instance, the predictor did not retain most neighboring interactions, which were strong IF values that differed greatly between leukemia sets.

Interestingly, we observed a significant difference between the average IF values of informative contacts in leukemias expressing MLL fusions compared to the *wt* protein (Figure 3B). Specifically, we found that a region downstream of *HOXA13* at the cluster 5’ end preferentially interacts with its 3’ part in *wt* MLL samples (Figure 3C, fragments 31 to 35). In contrast, more contacts were observed between the *HOXA11* gene (fragments 26 and 27) and the cluster, suggesting that these two regions are differentially regulated in leukemias expressing MLL fusions. This result was interesting in light of our previous report that differentiation of THP-1 promyelomonocytic leukemia cells into macrophages is accompanied by transcription repression of 5’ end genes and the formation of long-range contacts between the ends of the cluster [38]. Given that MLL fusions appear to alter organization, perhaps by modifying the chromatin at specific regions along the cluster, this result might also provide insight on how the fusions activate transcription. Whether DNA sequences at the *HOXA11* and *HOXA13* regions are important for the observed conformational changes is unclear but mapping of CTCF and cohesin by ChIP-seq shows that the two proteins bind to these regions (Figure 3C, bottom). CTCF and cohesin are known to form long-range interactions and it will be interesting to see whether their association with the chromatin or binding to each other to form loops are specifically targeted by MLL fusions.

**Figure 4:**
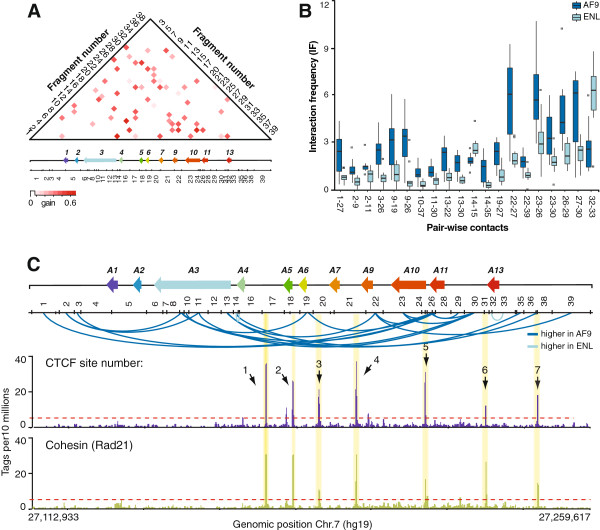
**Contacts throughout the *HOXA *cluster distinguish fusion subtypes. (A)** The information gain scores of *HOXA* contacts distinguishing MLL-AF9 from MLL-ENL leukemias are represented in heatmap form. The values are color-coded according to the scale shown at the bottom of the heatmap. Numbers on the left and right of heatmap identify *Bgl*II restriction fragments corresponding to the restriction pattern shown below the *HOXA* diagram. Intersecting column and row numbers identify DNA contacts. **(B)** Averaged interaction frequencies of 20 contacts with high information gain scores (IF values between MLL fusions are statistically different, *P* <0.01). Interaction frequencies in MLL fusion datasets are the averaged values from Additional file 4: Figure S2. Error bars represent the standard error of the mean. **(C)** Distribution of informative contacts for classification, and binding of CTCF and cohesin in THP-1 cells along the human *HOXA* cluster region examined. The y-axis shows the number of CTCF ‘Tags per 10 millions’ obtained by ChIP-seq after normalization against input, across the region characterized (x-axis). CTCF peaks are numbered from left to right (CTCF1 to 7).

In a similar manner, we looked for *HOXA* contacts showing the greatest informative differences between the MLL fusion subtype classes (Figure 4). For this, we measured the information gain value of each feature for the subtype prediction task and found 20 contacts with significant predictive value (Student t-test *P* value <0.01) (Figure 4A). In contrast to the predictive features of MLL leukemia types, the contacts distinguishing MLL-ENL from MLL-AF9 leukemias were distributed throughout the cluster (Figure 4B,C) and did not particularly cluster at sites bound by CTCF and cohesin. These were generally stronger in MLL-AF9 samples, except for 14-15 and 32-33 that were also identified as good predictors of *wt* MLL samples. We do not think that stronger contacts in MLL-AF9 can be explained simply by more expression of the cluster in either of the sample sets since each featured high and low expressers (Additional file 2: Table S1). Also, transcriptional activity does not appear to be a defining parameter in classification (see below and Additional file 5: Figure S4). Thus, we favor a model whereby different MLL fusions lead to distinct chromatin conformations by specifically recruiting proteins and modifying the chromatin at the cluster.

### *De novo* classification of MLL leukemia samples with 3D-SP

**Figure 5:**
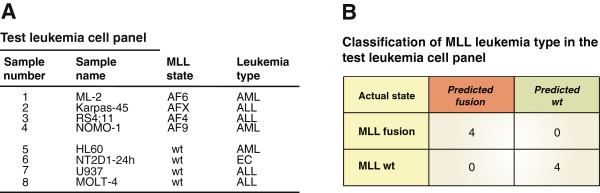
**3D-SP correctly classifies MLL leukemia types *de novo*. (A)** Leukemia cell panel used to test 3D-SP. Cell lines are organized by MLL status and leukemia type. **(B)** Classification results of the test leukemia cell panel by the 3D-SP trained to distinguish between MLL fusion and *wt* MLL (Figure 1C). Results shown are from *de novo* classification by the 3D-SP. Matthews Correlation Coefficient (MCC) = 1.0.

All the analyses with 3D-SP presented above were performed using a leave-one-out cross-validation approach and we wanted to confirm that the classifier would generalize to new samples. To this end, we generated 5C interaction maps for a test leukemia cell panel (Figure 5A and Additional file 6: Figure S3), and used the 3D-SP previously trained to distinguish between MLL *wt* and fusions with the training set (Figure 1C) to classify these data. The test leukemia set included leukemia cell lines expressing MLL-AF6, MLL-AFX, and MLL-AF4 and a new cell line expressing the MLL-AF9 fusion. Cell lines encoding *wt* MLL included AML, ALL, and the EC cell line NT2D1 induced with retinoic acid for 24 h. We added this sample because although it does not express an MLL fusion, the 3’ end genes are expressed and we expect the cluster to adopt an open configuration [52]. We found that 3D-SP classified the test leukemia cell lines expressing MLL fusions or *wt* MLL with perfect accuracy. Furthermore, 3D-SP also correctly classified five biological replicates of the MLL-AF9 expressing THP-1 samples produced in another study [55]. Even the EC sample expressing 3’ end *HOXA* genes was correctly classified suggesting transcription and opening of the cluster were not determining parameters in the classification by 3D-SP.

Indeed, transcription activity at the cluster did not seem to be a deciding factor in the classification since three of the four cell lines expressing MLL *wt* (HL60, U937, MOLT-4) expressed significant levels of *HOXA* genes, while one of the MLL fusion leukemia cell lines (Karpas-45) did not express the genes at all and yet, all were correctly classified (Additional file 2: Table S1). In fact, we found that 5C performed much better than gene expression when we compared SVM classification of a representative cell panel based on *HOXA9* expression (48%), all *HOXA* gene expression (62%) or on the 5C data (93%; Additional file 5: Figure S4B and C). Prediction based on gene expression was improved when a decision tree classifier was used instead of an SVM (*HOXA9*; 86%, all *HOXA*; 83%) but remained slightly lower than 5C classification with an SVM (Additional file 5: Figure S4D). Although our data do not definitively show that chromatin conformation is more robust than gene expression, 5C data do appear to contain additional information not present in gene expression datasets that improve classification. Together, these results provide the very first proof of principle that 3D chromatin organization of the *HOXA* cluster can be used to classify MLL fusion leukemia cell types.

## Conclusions

3D chromatin organization represents a type of higher-order transcription regulation mechanism used to control gene expression in a tissue-specific manner. Here, we provide evidence that chromatin conformation can be used to classify leukemia samples. We found that contacts from at least two regions along the *HOXA* cluster significantly contribute to the classification. The first region localizes at the 3’ end of the *HOXA11* gene and forms more contacts with the cluster when MLL fusions are expressed, while the second region lies downstream of the *HOXA13* gene and forms more contacts with the cluster in samples encoding the *wt* protein. These results suggest that these two regions are differently regulated in leukemias expressing MLL fusions, and one would expect that proteins interacting with one or more of these sequences would be responsible for these interactions. As described in the results section and discussed further below, we examined the changes in transcription among the various leukemias in our study, and found that they do not explain the differences in 5C. Interestingly however, we found that CTCF and cohesin are present at these interacting sites at least in the THP-1 cell line expressing MLL-AF9. CTCF and cohesin are known chromatin organizers [56] and it will be interesting to see whether differences in CTCF or cohesin binding at these sites play a role in the classification.

In the current model of genome structure-function, chromatin conformation and its molecular composition are thought to mutually affect each other to reach and propagate a given activity [33]. One argument, which could arise, therefore is that chromatin conformation accurately classifies leukemia samples because it reflects differences in transcription. However, comparison between classification with chromatin conformation and with gene expression suggests that at least in the case of MLL-fusion derived leukemias, the information provided by the chromatin organization does not simply reflect its transcriptional state. For example, samples that express high levels of 5’ end *HOXA* genes might all classify together because chromatin is likely to be more open in that part of the cluster. We show that this is not the case first by demonstrating that cell samples expressing different levels of *HOXA* genes classify properly in the same groups, and secondly, by demonstrating that classification is at least comparable or better with chromatin conformation than gene expression. What the classification results suggest is that as a chromatin modifier, the MLL *wt* and MLL fusion proteins differently affect the molecular composition and conformation of the chromatin in a manner that is at least partly independent of its effect on transcription activity. In turn, our results show that chromatin organization is not merely tissue-specific but can also differ when different diseases affect a given cell type. Long-range transcription regulation might therefore be specifically altered in human diseases, and sometimes even significantly contribute to pathologies.

In the future, it will be interesting to determine whether expanding the 5C analysis to other MLL fusion target domains can predict leukemia types and subtypes with near-perfect accuracy. Identifying the type and subtype of the leukemia is the most important factor in defining a treatment course, and for this reason it will also be interesting to see whether chromatin conformation can accurately classify samples collected from the blood of patients, and predict overall survival. With the constant improvement of deep sequencing methods and of protocols capturing genome organization [57,58], identifying *bona fide* chromatin conformation signatures useful in the clinic is becoming increasingly feasible. The clinical importance of biomarkers for diagnosis, therapy selection, and for screening and monitoring disease progression is already appreciated in the treatment of many cancer types [59]. The identification of robust biomarkers will be important in directing patient care towards a customized setting and will require a greater overall understanding of genome regulation including how it is organized in the nuclear space.

## Materials and methods

### Ethical approval

No ethical approval was needed for this study.

### Cell culture and description of the 5C datasets

All experiments presented in this study were performed using actively growing cells (log-phase) as defined by growth curves. All cells were grown at 37°C in 5% CO_2_ atmosphere. A complete description of all the cell samples can be found in Additional file 1: Supplementary Materials and methods. The normalized 5C data in matrix format are found in Additional file 7.

### Quantitative real-time polymerase chain reaction (RT-qPCR)

*HOXA* gene expression was measured by quantitative real-time polymerase chain reaction and is shown relative to actin. The RNeasy^©^ Mini kit (Qiagen) was used to extract total RNA from the samples as described by the manufacturer. The samples were then treated with DNAseI (NEB), and re-purified on Qiagen columns. Two micrograms of the resulting total RNA was used in each reverse transcription reaction with the Superscript^®^ III reverse transcriptase kit (Invitrogen™) and oligo (dT)_20_. The SsoFast™ EvaGreen^©^ Supermix (Bio-Rad) was used to quantify the samples by RT-qPCR with a Bio-Rad CFX96™ (C1000™ series) real-time system. Quantification was performed by two-fold serial dilutions of total cDNA. The size and specificity of amplified products was verified on agarose gels containing 0.5 μg/mL ethidium bromide and/or by verifying the melting temperatures of PCR amplicons. An AlphaImager^©^ HP coupled to a 12-bit digital camera and equipped with the AlphaView^®^ image acquisition and analysis software (version 3.0; Alpha Innotech Corporation) was used to document and analyze the gels. All RT-qPCR primer sequences used in this study were previously described [38], and their sequences are also available on our website [60].

### Chromosome conformation capture (3C) and 3C-carbon copy (5C)

The chromosome conformation capture (3C) and 3C-carbon copy (5C) techniques were used as previously described to characterize the chromatin organization of a region containing the *HOXA* gene cluster [38,51]. The experimental design and the procedure used to generate our 3C and 5C datasets are described in detail in Additional file 1: Supplementary Materials and methods.

### Interaction frequency (IF) normalization

We modified the previously published approach to normalize interaction frequency data [51] to account for the amount of DNA hybridized onto arrays (hybridization efficiency) and improve correction for primer pair efficiencies. This approach is described in detail in Additional file 1: Supplementary Materials and methods, and is illustrated in Additional file 3: Figure S1.

### Support vector machine implementation

A support vector machine (SVM) classifier [53] was implemented using the open-source Weka Java package [61,62]. The SVM hyperparameters (notably the soft margin penalty and the RBF width) were fixed for all experiments and the SVM used a polynomial kernel (K (x, y) = < x, y > ^p^) and was trained using sequential optimization minimization, with 1-vs-1 pairwise classification for multi-class problems [63,64]. Each 5C dataset was represented as a single vector of normalized IF values obtained by measuring pairwise chromatin interactions *in vivo*. The SVM was trained and evaluated using a leave-one-out cross-validation approach to obtain a classifier with a maximum-margin hyperplane in the transformed feature space. The predictive power of individual features was evaluated using the information gain score (also computed using Weka), which measures the reduction of the entropy of the class distribution when the feature is considered. Contacts with large information gains confer the highest amount of predictive power. Informative contacts were selected by determining if the IF values were statistically different between sample types. This was done by calculating *P* values using a two-sided Student’s t-Test in R [65]. The two samples (MLL fusions *vs. wt*, or MLL-AF9 *vs.* MLL-ENL) t-test was performed for each feature independently, and features that were retained had reported *P* values below a threshold of 0.01 (*P* <0.01).

The Matthews Correlation Coefficient (MCC) was calculated as a measure of the quality of the classifications performed by the SVM, with values close to +1 indicating perfect prediction, values close to 0 indicating random prediction, and values close to -1 indicating complete disagreement between the prediction and the observation. The MCC statistic was calculated from the confusion matrix as:

$$\begin{array}{l} \text{MCC}=\frac{\left(\left(\mathrm{TP}\;x\;\mathrm{TN}\right)-\left(\mathrm{FP}\;x\;\mathrm{FN}\right)\right)}{\text{sqrt}\;\left(\left(\mathrm{TP}+\mathrm{FP}\right)\times \left(\mathrm{TP}+\mathrm{FN}\right)\times \left(\mathrm{TN}+\mathrm{FP}\right)\times \left(\mathrm{TN}+\mathrm{FN}\right)\right)}\\ \text{where}\;\mathrm{TP}:\text{True}\;\text{Positives},\mathrm{TN}:\text{True}\;\text{Negatives},\mathrm{FP}:\text{False}\;\text{Positives},\\ \;\mathrm{FN}:\text{False}\;\text{Negatives} \end{array}$$

### ChIP-seq

Chromatin immunoprecipitation was conducted as described previously [55] with 5 μL of antibodies against CTCF (Millipore, cat. no. 07–729) or Rad21 (Abcam, cat. no. ab992). For Rad21 samples, the BARCODE adaptors were used for generating the BARCODE ChIP-seq libraries (BARCODE adaptor [66]). ChIP-seq libraries were sequenced with an Illumina Genome Analyzer (GAiiX) DNA sequencer (36 bps reads, CTCF and WCE) and Illumina Hiseq2000 (51 bps reads, Rad21) at the Génome Québec Innovation Centre [67].

### ChIP-seq data analysis

For BARCODE ChIP-seq libraries (Rad21), FASTX-Toolkit (version 0.0.13) [68] was used to split the reads according to the specific nucleotide barcode in first 4 bp of sequence reads. Before mapping to the genome, the first 4 bps barcode nucleotide of each read were trimmed. Sequence reads with low quality in 3’ ends were trimmed to 25 bps (one lane of sequence reads in Rad21 samples). Sequence reads (Fastq format) were aligned to the human genome (UCSC hg19) using the Bowtie program [69]. One mismatch was allowed to the unique mapped reads (option: -v 1 -m 1). Peak calling was performed with the HOMER program [70] and WCE data was used as control. The total mapped tags in each sample were normalized to ‘Tags per 10 millions’ reads. The CTCF or Rad21 binding regions with two-fold (CTCF) or four fold (Rad21) over in both control (whole cell extract) and local region (10 kbp) were identified by HOMER program (FDR <0.001 (‘findpeak’ command)). The data was visualized in the hg19 genome version on the UCSC browser.

### Databases and URLs

The data and source code of our software are available through our website with instructions at the following address: [71]. The source code of our software is also attached to this manuscript for archival purpose only (Additional file 8). The 5C and ChIP-seq data for CTCF and Rad21 can be downloaded from the Gene Expression Omnibus (GEO) website [72].

## Abbreviations

3C: Chromosome conformation capture; 5C: 3C-carbon-copy; SVM: Support vector machine.

## Competing interests

The authors declare that they have no competing interests.

## Authors’ contributions

MR designed, developed, and carried out the computational analysis, and helped to draft the manuscript. MAF participated in designing the 5C experiments, produced part of the 5C datasets, and participated in the mRNA quantification by real-time quantitative PCR. JC carried out part of the 5C experiments, participated in the quantification of mRNAs by real-time quantitative PCR, and helped to draft the manuscript. XQDW participated in generating part of the 5C experiments, and in the quantification of mRNAs by real-time quantitative PCR. HM performed the ChIP-seq experiments and their analysis. MB participated in the design and coordination of the study, and helped to draft the manuscript. JD conceived the study, participated in its design and coordination, and drafted the manuscript. All authors read and approved the final manuscript.

## Supplementary Material

Additional file 1 **Supplementary Materials and methods, Supplementary Figure legends, and Supplementary References.** The Supplementary Materials and methods contain a detailed description of the cell lines analyzed in this study. It also describes the 3C and 5C protocols used to capture chromatin organization, and the computational approach developed to calculate normalized interaction frequencies (IFs). This file also contains the figure legends for (Additional file 3: Figures S1, Additional file 4: Figures S2, Additional file 5: Figures S4, and Additional file 6: Figures S3 respectively), as well as References relevant to Additional file 1. Click here for file

Additional file 2: Table S1 *HOXA* gene expression in leukemia cell samples used in this study. This table contains the mRNA quantification of all *HOXA* genes by RT-qPCR in cell lines used to compare classification by gene expression and chromatin conformation (Additional file 5: Figure S4). Click here for file

Additional file 3: Figure S1 Workflow of 5C data processing, normalization, and conversion into interaction frequencies. This figure illustrates our approach to calculate normalized IFs from 5C data. This method and Additional file 3: Figure S1 are described in detail in (Additional file 1: Supplementary Materials and methods and Additional file 3: Figure S1). Click here for file

Additional file 4: Figure S2 5C datasets generated for the 3D-SP training set. This figure shows the 5C data of the cell samples from the training set in the form of heatmaps as described in Additional file 4: Figure S2 (Additional file 1). Click here for file

Additional file 5: Figure S4 3D-SP performs better than gene expression to classify MLL leukemia types. This figure compares classification of leukemia cell samples using *HOXA* gene expression and *HOXA* chromatin organization. The figure is described in the Additional file 5: Figure S4 in Additional file 1. Click here for file

Additional file 6: Figure S3 5C datasets generated for the 3D-SP test set. This figure shows the 5C data of the cell samples from the test set in the form of heatmaps as described in Additional file 6: Figure S3 (Additional file 1). Click here for file

Additional file 7 **This file contains the normalized 5C data in matrix format of all the samples produced for this study.** These files can be uploaded directly in the my5C platform (http://my5c.umassmed.edu/welcome/welcome.php) [73]. Click here for file

Additional file 8 This file contains the source code for all the software used in this study. Click here for file
