# Supplementary material for: Classifying leukemia types with chromatin conformation data
Source: Genome Biol. 2014 Apr 30;15(4):R60. doi: 10.1186/gb-2014-15-4-r60 (PMC4038739; doi:10.1186/gb-2014-15-4-r60)
Supplement: Additional file 1 — Supplementary Materials and methods, Supplementary Figure legends, and Supplementary References. The Supplementary Materials and methods contain a detailed description of the cell lines analyzed in this study. It also describes the 3C and 5C protocols used to capture chromatin organization, and the computational approach developed to calculate normalized interaction frequencies (IFs). This file also contains the figure legends for (Additional file 3: Figures S1, Additional file 4: Figures S2, Additional file 5: Figures S4, and Additional file 6: Figures S3 respectively), as well as References relevant to Additional file 1. [file gb-2014-15-4-r60-S1.docx]

**Supplementary Materials and methods**

**Cell culture and description of the 5C datasets**

*AF9-A-1 and AF9-A-2*

AF9-A cells express the MLL-AF9 fusion protein, are karyotypically normal, and were derived from an *in vivo* model that recapitulates the initiation and progression of acute monocytic leukemia (AML) [[1](#_ENREF_1), [2](#_ENREF_2)]. Briefly, human cord blood transduced with an MSCV retrovirus encoding human MLL-AF9 was injected intra-femorally into NOD/SCID mice after various days in culture. Bone marrow cells were harvested and cultured *ex-vivo* to isolate individual clones. Each clone was cultured in alpha-MEM (Minimum Essential Media) containing glutamine, ribonucleosides and deoxyribonucleosides (Gibco, cat. no. 12571-071), 20% fetal bovine serum (FBS; HyClone Premium Serum), 5% human plasma (Stem Cell Technologies peripheral blood, cat. no. PB021F), and a growth factor cocktail containing the following human recombinant factor: 100 ng/ml rhSCF (Gibco, cat. no. PHC2111), 10 ng/ml rhIL-3 (Gibco, cat. no. PHC0034), 5 ng/ml rhIL-7 (Gibco, cat. no. PHC0075), 5 ng/ml rhFLT3 (Gibco, cat. no. PHC9414). The media was not supplemented with antibiotics, and the human plasma was filtered through a 0.22 *µ*M membrane before adding to the media. The growth factor cocktail was prepared as a mixture and stored in PBS containing 0.5% BSA (Sigma, cat. no. A9418-5G) at -20°C. AF9-A is one of these clones, and AF9-A-1 and AF9-A-2 are biological replicates. These cells were kindly provided by Dr. J. Dick (University of Toronto, Canada).

*THP1-A-1, THP1-B-1, THP1-C-1, THP1-D-1, and THP1-E-1*

THP1 are promyelomonocytic cells derived from a 1-year-old male suffering from AML at relapse in 1978 [[3](#_ENREF_3)]. The THP1 cell line used in this study is a subclone (THP1.5) selected for its ability to differentiate homogenously in response to treatment with phorbol 12-myristate 13-acetate (PMA)[[4](#_ENREF_4)]. These cells encode a translocation resulting in the expression of an MLL-AF9 fusion protein. The THP1-A to -E datasets are from a differentiation time-course with PMA [[5](#_ENREF_5)]. THP1-A-1 are untreated cells collected at the 0h time point. THP1-B-1, THP1-C-1, THP1-D-1, and THP1-E-1 are treated with 30 ng/ml PMA, and were collected at the 4 h, 12 h, 48 h, and 96 h time points, respectively. These cells were grown in Roswell Park Memorial Institute medium (RPMI 1640; Invitrogen™) containing 10% FBS (HyClone), 50 mM 2-mercaptoethanol (Invitrogen™), 1 mM sodium pyruvate (Invitrogen™), 10 mM HEPES (Invitrogen™), and 1% penicillin-streptomycin (Invitrogen™). The THP1.5 clone was obtained from Dr. Y. Hayashizaki from the RIKEN Genome Exploration Research Group (RIKEN Yokohama Institute, Japan).

*THP1-E-2, THP1-F-1, and THP1-F-2*

The THP1-E-2 sample is THP1 cells treated with 30 ng/ml PMA for 96 h, and is a biological replicate of THP1-E-1. THP1-F-1 and THP-F-2 are control THP1 cells treated with 0.003% DMSO for 96 h. THP1-F-1 is the control of THP1-E-2 and these datasets were previously published [[6](#_ENREF_6)]. THP1-F-2 is a biological replicate of THP1-F-1. Cells were grown as described above.

*ENL-A-1, ENL-A-2, ENL-A-3, ENL-A-4*

ENL-A cells express the MLL-ENL fusion protein and are karyotypically normal. These cells derive from an *in vivo* model that recapitulates the initiation and progression of acute lymphoblastic leukemia (ALL) as described above for AF9-A cells, except that the retrovirus encoded the human MLL-ENL protein. ENL-A is one of these clones and ENL-A-1, ENL-A-2, and ENL-A-3 are biological replicates, whereas ENL-A-4 is a technical replicate of ENL-A-2. Specifically, the ENL-A-4 dataset is from a new 3C library prepared from a cell pellet fixed on the same day as ENL-A-2). These cells were grown as described for AF9-A, and kindly provided by Dr. J. Dick (University of Toronto, Canada).

*ENL-B-1, ENL-B-2, ENL-B-4*

ENL-B cells express the MLL-ENL fusion protein and are karyotypically normal. These cells are as described for ENL-A, but are from a different clone. ENL-B-2 is a technical replicate of ENL-B-1, whereas ENL-B-4 is a biological replicate. Specifically, the ENL-B-2 dataset is from a new 3C library prepared from a cell pellet fixed on the same day as the ENL-B-1. These cells were grown as described for AF9-A, and kindly provided by Dr. J. Dick (University of Toronto, Canada).

*HB1119*

HB1119 cells encode a translocation between the MLL and ENL genes (t(11;19)). The cell line was established from a patient with B cell precursor ALL, and express an MLL-ENL fusion protein from one allele. Cells were grown in RPMI 1640 (Wisent) supplemented with 10% FBS (HyClone), 1mM L-glutamine (Gibco) and 10% penicillin-streptomycin (Invitrogen™). This dataset was previously published [[7](#_ENREF_7)]. This cell line was a kind gift from Dr. M. Cleary (Stanford University, USA).

*KOPN8-1 and KOPN8-2*

KOPN8 cells are established from the peripheral blood of a 3-month-old female with B-cell precursor ALL in 1977 [[8](#_ENREF_8)]. These cells carry a translocation resulting in the expression of an MLL-ENL fusion protein. KOPN8-2 is a technical replicate of KOPN8-1. Specifically, the KOPN8-1 and KOPN8-2 datasets are from two separate 5C libraries that were prepared from the same 3C library. Cells were grown in RPMI 1640 (Gibco) supplemented with 10% FBS (Fisherbrand) and 1% penicillin-streptomycin (Invitrogen™). These cells were obtained from the Leibniz Institute DSMZ-German Collection of Microorganisms and Cell Cultures (cat. no. ACC-552).

*NB4-1 and NB4-2*

NB4 cells are established from the bone marrow of a 23-year-old female with acute promyelocytic leukemia (APL) in second relapse in 1989 [[9](#_ENREF_9)]. These cells encode the wild-type MLL protein. NB4-2 is a technical replicate of NB4-1. Specifically, the NB4-2 dataset is from a new 3C library prepared from a cell pellet fixed on the same day as NB4-1. Cells were grown in RPMI 1640 medium (Gibco), supplemented with 10% FBS (Wisent), 1 mM L-glutamine (Invitrogen™), and 1% penicillin-streptomycin (Invitrogen™). These cells were a gift from Dr. J. Teodoro (McGill University, Canada).

*NALM6-1, NALM6-3, NALM6-4, NALM6-5*

NALM6 cells are established from the peripheral blood of a 19-year-old man with ALL in relapse in 1976 [[10](#_ENREF_10)]. NALM6 cells express the wild-type MLL protein. NALM6-1, NALM6-4, NALM6-5 are biological replicates, whereas NALM6-3 is a technical replicate of NALM6-1. Specifically, the NALM6-1 and NALM6-3 datasets are from two separate 5C libraries that were prepared from the same 3C library. Cells were grown in RPMI 1640 medium (Wisent), supplemented with 10% FBS (HyClone), and 1% penicillin-streptomycin (Gibco). These cells were obtained from the Leibniz Institute DSMZ-German Collection of Microorganisms and Cell Cultures (cat no. ACC-128).

*Jurkat*

Jurkat cells were established from a 14-year-old with ALL at first relapse in 1976 [[11](#_ENREF_11)]. These cells express the wild-type MLL protein. Cells were grown in RPMI 1640 (Gibco) supplemented with 10% FBS (Fisherbrand), and 1% penicillin-streptomycin (Invitrogen™). These cells were a gift from Dr. J. Teodoro (McGill University, Canada).

*NT2D1-1, NT2D1-2, and NT2D1-3*

The NT2/D1 cell line (NTERA-2 cl. D1) was derived by cloning from NTERA-2 cell line, which originates from a lung metastasis in a 22-year-old Caucasian male [[12](#_ENREF_12)]. NT2D1 is a pluripotent human testicular embryonal carcinoma cell line that can be induced to differentiate into neuroectodermal lineages following treatment with retinoic acid (RA). These cells express the wild-type MLL protein. NT2D1-1, NT2D1-2, and NT2D1-3 are biological replicates. These cells were obtained from ATCC (American Type Culture Collection, cat. no. CRL-1973), and cultured in Dulbecco’s Modified Eagle’s Medium (DMEM; Gibco) supplemented with 10% FBS (HyClone) in the presence of 1% penicillin–streptomycin (Invitrogen™; 'complete DMEM') as described previously (Ferraiuolo *et al*, 2010).

*ML-2*

ML-2 cells were established from the peripheral blood of a 26-year-old male at the diagnosis of AML in 1978 [[13](#_ENREF_13)]. The cell line carries a translocation resulting in the expression of an MLL-AF6 fusion protein. Cells were grown in RPMI 1640 (Gibco) supplemented with 10% FBS (Fisherbrand), and 1% penicillin-streptomycin (Invitrogen™). These cells were obtained from the Leibniz Institute DSMZ-German Collection of Microorganisms and Cell Cultures (cat. no. ACC-15).

*Karpas-45*

The Karpas-45 cell line was established from the bone marrow of a 2-year-old male at the diagnosis of ALL prior to therapy in 1972 [[14](#_ENREF_14)]. The cell line expresses an MLL-AFX fusion protein. Cells were grown in RPMI 1640 (Gibco) supplemented with 20% FBS (Fisherbrand) and 1% penicillin-streptomycin (Invitrogen™). These cells were obtained from the Leibniz Institute DSMZ-German Collection of Microorganisms and Cell Cultures (cat. no. ACC-105).

*RS4;11*

RS4;11 cells were derived from the bone marrow of a 32-year-old female with ALL in first relapse [[15](#_ENREF_15)]. These cells encode a translocation resulting in the expression of an MLL-AF4 fusion protein. Cells were grown in RPMI 1640 (Gibco) supplemented with 10% FBS (Fisherbrand) and 1% penicillin-streptomycin (Invitrogen™). These cells were obtained from ATCC (American Type Culture Collection, cat. no. CRL-1873).

*NOMO-1*

NOMO-1 cells were established from a 31-year-old female with AML at second relapse [[16](#_ENREF_16)]. These cells carry a translocation resulting the expression of the MLL-AF9 fusion protein. Cells were grown in RPMI 1640 (Gibco) supplemented with 10% FBS (Fisherbrand), and 1% penicillin-streptomycin (Invitrogen™). These cells were obtained from the Leibniz Institute DSMZ-German Collection of Microorganisms and Cell Cultures (cat. no. ACC-542).

*THP1-A-2, THP1-B-2, THP1-C-2, THP1-D-2, and THP1-E-3*

THP1-A-2, THP1-B-2, THP1-C-2, THP1-D-2, and THP1-E-3 are technical replicates of the PMA-induced differentiation time course collected at 0 h, 4 h, 12 h, 48 h, and 96 h, respectively.

*HL60*

The HL60 cell line was derived from the peripheral blood of a 35-year-old female AML in 1976 [[17](#_ENREF_17)]. This cell line expresses the wild-type MLL protein. Cells were grown in RPMI 1640 (Gibco) supplemented with 10% FBS (Fisherbrand), and 1% penicillin-streptomycin (Invitrogen™). These cells were a gift from Dr. J. Teodoro (McGill University, Canada).

*NT2D1-24h*

The NT2D1 cell line differentiates along neuroectodermal lineages after exposure to retinoic acid (RA) [[18](#_ENREF_18)]. The NT2D1-24h sample is composed of cells cultured in complete DMEM containing 10 µM RA for 24 hours.

*U937*

The U937 cell line was established from the pleural effusion of a 37-year-old man with generalized diffused histiocytic lymphoma in 1974 [[19](#_ENREF_19)]. This cell line encodes the wild-type MLL protein. Cells were grown in RPMI 1640 (Gibco) supplemented with 10% FBS (Fisherbrand), and 1% penicillin-streptomycin (Invitrogen™). These cells were kindly provided by the Laboratory of Therapeutic Development, Goodman Cancer Research Centre, McGill University, Canada.

*MOLT-4*

MOLT-4 cells are derived from the peripheral blood of a 19-year-old man with ALL in relapse in 1971 [[20](#_ENREF_20)]. These cells encode the wild-type MLL protein. Cells were grown in RPMI 1640 (Gibco) supplemented with 10% FBS (Fisherbrand), and 1% penicillin-streptomycin (Invitrogen™). These cells were a gift from Dr. J. Teodoro (McGill University, Canada).

**Chromosome conformation capture (3C) and 3C-carbon copy (5C)**

Briefly, 3C libraries were prepared by fixing approximately 1X10^7^ cells in the presence of 1% formaldehyde. The samples were then digested overnight with the *Bgl*II restriction enzyme (NEB), and ligated with T4 DNA ligase for 2 hours at 16°C under dilute conditions favoring ligation events between cross-linked fragments. Crosslinking was reversed using Proteinase K and the resulting 3C libraries were purified by phenol/chloroform extraction. For quantification and quality control, 3C libraries were titrated by PCR by measuring contacts in a control gene desert region (ENCODE ENr313) as described previously [[21](#_ENREF_21)]. Conventional 3C analysis was used to measure contacts for Figure S5, and is a described previously with primers available upon request [[22](#_ENREF_22)].

To generate the 5C libraries, a pool of 5C primers described in [[6](#_ENREF_6)] was added to individual 3C libraries in a final volume of 10 μl of annealing buffer (20 mM Tris-acetate pH 7.9, 50 mM potassium acetate, 10 mM magnesium acetate, and 1 mM dithiothreitol), and to final individual primer concentrations of 20 nM. 5C primers were annealed to 3C templates overnight at 48°C, and ligated the following day by adding 20 μl of ligation buffer containing 10 units of Taq DNA ligase for 1 h at 48°C (NEB; 25 mM Tris-HCl pH 7.6, 31.25 mM potassium acetate, 12.5 mM magnesium acetate, 1.25 mM NAD, 12.5 mM dithiothreitol, 0.125% Triton X-100). The reactions were terminated by a 10 minute incubation at 65ºC and amplified by PCR with the T7 (TAATACGACTCACTATAGCC) and 5’-Cy3-labelled T3 (TATTAACCCTCACTAAAGGGA) primers, which are complementary to the common forward and reverse tail sequences of 5C primers, respectively. Unincorporated primers and other contaminants were removed from 5C libraries using the MinElute Reaction Cleanup kit (Qiagen®) as recommended by the manufacturer.

The 5C primer pool contained alternating forward and reverse 5C primers complementary to consecutive *Bgl*II fragments in the *HOXA* cluster and control gene desert ENr313 regions (hg19|chr7:27,112,593-27,254,038) and (hg19|chr16:62,276,449-62,776,448), respectively. This 5C experimental design considers a region of 141 kbp on human chromosome 7 containing the *HOXA* cluster, and a region of 33 kbp on human chromosome 16 containing the control ENr313 gene desert region. The *HOXA Bgl*II fragments characterized in this study were numbered from 1 to 39. Note that a 5C primer against fragment 2 was not included during the production of 5C libraries because this region contains a repetitive sequence. The regions spanning fragments 18 and 20 each contain two *Bgl*II fragments. The two excluded fragments (18b and 20b) were removed from our analysis because the corresponding 5C primers were homologous to each other. This library design measured up to 756 different pair-wise interactions. The 5C primer sequences used in this analysis were previously described [[6](#_ENREF_6)] and are also available on our website [[23](#_ENREF_23)].

The 5C libraries were quantified by 5C-chip, using custom designed microarrays (Roche Diagnostics), as described in [[6](#_ENREF_6)]. The 5C arrays contained oligonucleotide probes corresponding to the sense strand of all possible 5C ligation products within the four human *HOX* clusters and the control ENr313 gene desert region. Each predicted contact was featured 8 times by replicates of increasing lengths (30, 36, 38, 40, 42, 44, 46, and 48 nucleotides). Each set of eight probes was complementary to the same ligation product target and should therefore display monotonically increasing signal intensity with increase probe length from the stronger hybridization of longer complementary sequences. As per the manufacturer’s guidelines, approximately 100 ng of 5C library were individually hybridized to arrays using the NimbleGen CGH Hybridization kit [[24-26](#_ENREF_24)]. 5C arrays were scanned with a DNA microarray scanner (Agilent Technologies, model G2505) at 5 μm resolution, the raw data were extracted from the images using the NimbleScan 2.6 software (NimbleGen Systems, Inc.), and specific features were extracted with our 'ArrayQC' software [[6](#_ENREF_6)].

**Interaction frequency (IF) normalization**

In our method, the raw microarray signal intensity values are first extracted using the ‘ArrayQC’ program as previously described [[6](#_ENREF_6)]. A filter is then applied to the signals from the *HOXA* region to remove any signal above 40,000, which we consider as saturated (the detection limit of the scanner is 65,536). For the signals from the gene desert region, we only retain those from neighbor and near-neighbor fragment pairs, with a maximum gap between neighbors of 3 indices. We remove all other contacts because as they derive from the interaction of distal fragments, their signals tend to be weak and not reliable. The gene desert signals retained are then used to calculate the normalization factors as follows: the median intensity value is calculated for each probe length in each experiment, and the mean median value is determined for each probe length across all experiments. These mean median values (one per probe length) are then used as the ‘target’ values in the normalization process. For each experiment the ratio of the average signal value to the ‘target’ value for each probe length is calculated and saved as the normalization factor, such that there is a total of eight normalization factors (one per probe length) for each experiment. These normalization factors are subsequently applied by multiplying the values from each probe in the gene desert and the *HOXA* regions by the normalization factor corresponding to that experiment and to the given probe length. This normalization step yields average gene desert signals for each probe lengths that are equal across all of the experiments, such that a background and trend filter can be applied next. The background filter functions as previously described [[6](#_ENREF_6)], and uses the signal from the 30 nt length probe as a measure representative of the background signal and excludes any of the longer length probes that are not greater than a specified percentage of its value (150%). The background signal is then subtracted from the remaining values, and a trend filter is applied that enforces the property that signals should monotonically increase with respect to probe length. If any values do not pass this test, then the longest properly behaved subsequence is identified and the remainder of the values are excluded. The remaining values are then normalized for primer pair efficiency using a control 3C library produced from bacterial artificial chromosome (BAC) to obtain the final IF values as detailed below and previously described [[27](#_ENREF_27)].

*5C product (primer pair) hybridization efficiency normalization*

5C products derive from the ligation between 5C primer pairs, which vary in base composition and can hybridize with different efficiencies onto arrays. We normalize 5C experiments for primer pair efficiencies with a control 5C library. The control 5C library contains equimolar ratios of all possible 5C products under study and is hybridized onto a custom microarray in the same manner as a cellular 5C library described above. The control 5C library dataset is then processed, and used to calculate interactions frequencies (IF) as described below.

To generate control 5C libraries for our study, we first prepared control 3C libraries using BAC clones as described previously [[22](#_ENREF_22), [28](#_ENREF_28)]. Our libraries contained the four human *HOX* clusters and the ENr313 gene desert region and were made using the BAC clones RP11-1132K14, CTD-2508F13, RP11-657H18, RP11-96B9, RP11-197K24, CTD-2594L23, and RP11-1132K14 purchased from Invitrogen^TM^. These clones were mixed, digested with *BglI*I and randomly ligated in solution with T4 DNA ligase to yield all possible ligation junctions in equimolar ratios. Control 5C libraries were then prepared and hybridized as described for the cellular 5C libraries. To normalize the ensemble of 5C experiments from our study in a consistent manner, we generated a set of six control 5C datasets, which we combined to create a single control 5C normalization reference called 'master BAC'. The control 5C datasets were produced in duplicates from three independent control 3C libraries produced by three lab members. For each control 5C dataset, the raw signal intensities were extracted using the ArrayQC program, and normalized for DNA hybridization efficiency using an approach similar to the one described above for cellular 5C libraries. Instead of using the signals from the gene desert region to calculate the normalization factors for DNA hybridization efficiencies, we used the signals from the *HOXA* region since control 5C datasets contain equimolar quantities of all possible ligation products. Similarly to the method described above, the mean median values per-probe length were used as the ‘target’ values, and the normalization values were applied such that the resulting per-probe length average signal was equal across all of the experiments. The ‘master BAC' was then constructed by taking the average value for each probe pair across the ensemble of normalized BAC experiments, and the single resulting dataset was cleaned using the 30 nt length probe as a measure of background (150% cutoff used as above and previously described in [[6](#_ENREF_6)]). The resulting control 5C product signals were used to calculate the IF values by dividing the signals from each 5C experiment by their corresponding entry in the ‘master BAC'. Finally, we applied a cleaning filter whereby entries with standard deviation values larger than a specified percentage (75%) of their respective IF value are excluded.

**Supplementary Figure legends**

**Figure S1. Workflow of 5C data processing, normalization, and correction for primer pair efficiency.**

**(A)** Workflow of raw data processing and normalization. Raw 5C microarray data are filtered and normalized into 3 steps shown from top to bottom (red-orange-yellow-green). The raw 5C data from the *HOXA* cluster and a control gene desert region (highlighted in red) are first filtered to remove any measurements that are saturated using a saturation filter of 40,000. This first step differs between the *HOXA* test locus and the control gene desert: for the *HOXA* signals, all remaining points are retained whereas for the gene desert, only signals from neighbors and next-neighbor fragment pairs in the linear genome are selected (highlighted in orange; see Supplementary Materials and methods for details). A median normalization method (described in Materials and methods) is then applied (highlighted in yellow). The normalized *HOXA* data are then filtered to remove the background signal from the data and any measurement that does not follow a trend of monotonically increasing signals with increasing probe length (highlighted in green). **(B)** A control library is required to correct for primer pair efficiency. Conversion of normalized 5C signals into interaction frequency values is achieved by dividing the normalized 5C data processed as in **(A)** by a 'Master BAC' control library (highlighted in blue) as described in the Supplementary Materials and methods section. 5C microarray data are plotted as box plots, with signal intensity on the *y-axis* and probe half-length (nt) on the *x-axis*.

**Figure S2. 5C datasets generated for the 3D-SP training set.**

**(A)** Chromatin contacts at the *HOXA* cluster region in samples expressing MLL fusion proteins are represented in heatmap form. Pair-wise interaction frequencies were normalized as described in Figure S1, and are color-coded according to the scale shown on the bottom left. Samples are identified by their names above the heatmaps. Numbers above and on the right of each heatmap identify *Bgl*II restriction fragments corresponding to the restriction pattern shown below the *HOXA* diagram in Figure 1A. Intersecting column and row numbers identify DNA contacts. **(B)** Chromatin contacts at the *HOXA* cluster region in samples expressing the *wt* MLL protein are presented in the form of two-dimensional heatmaps as described in panel **(A)**.

**Figure S3. 5C datasets generated for the 3D-SP test set.**

5C interaction maps of the *HOXA* cluster region in samples used to test the accuracy of 3D-SP at classifying new MLL leukemia samples are represented in heatmap form. Pair-wise interaction frequencies were normalized as described in Figure S1, and are color-coded according to the scale shown on the bottom left. Samples are identified by their names above the heatmaps. Numbers above and on the right of each heatmap identify *Bgl*II restriction fragments corresponding to the restriction pattern shown below the *HOXA* diagram in Figure 1A. Intersecting column and row numbers identify DNA contacts.

**Figure S4. 3D-SP performs better than gene expression to classify MLL leukemia types. (A)** Quantitative real-time PCR analysis of the *HOXA9* gene expression in the cell lines used in this study. Steady-state mRNA levels were normalized relative to actin (*y-axis*). Sample names, their MLL status and the leukemia type are indicated below the *x-axis*. Each histogram value is the average of at least three PCR reactions and the error bars represent the standard error of the mean.  **(B)** Leukemia cell panel used to compare classification using 5C data and gene expression. Cell lines are organized by leukemia type and MLL status. **(C)** Comparison of the overall performance of *HOXA9* expression, all *HOXA* gene expression, and 3D-SP trained with samples from panel **B** at classifying leukemia samples expressing MLL fusion or the *wt* protein listed in **(B)**. **(D)** Classification results of the top two classification methods. SVM results are from leave-one-out cross-validation analysis using the data set shown in panel **B**. The Matthews Correlation Coefficient of the decision tree classification is equal to 0.69. The Matthews Correlation Coefficient of the 3D-SP classification is equal to 0.85.

**Supplementary References**

1. Barabe F, Kennedy JA, Hope KJ, Dick JE: **Modeling the initiation and progression of human acute leukemia in mice.** *Science* 2007, **316:**600-604.

2. Kennedy JA, Barabe F: **Investigating human leukemogenesis: from cell lines to in vivo models of human leukemia.** *Leukemia* 2008, **22:**2029-2040.

3. Tsuchiya S, Yamabe M, Yamaguchi Y, Kobayashi Y, Konno T, Tada K: **Establishment and characterization of a human acute monocytic leukemia cell line (THP-1).** *Int J Cancer* 1980, **26:**171-176.

4. Suzuki H, Forrest AR, van Nimwegen E, Daub CO, Balwierz PJ, Irvine KM, Lassmann T, Ravasi T, Hasegawa Y, de Hoon MJ, et al: **The transcriptional network that controls growth arrest and differentiation in a human myeloid leukemia cell line.** *Nat Genet* 2009, **41:**553-562.

5. Rousseau M, Crutchley JL, Miura H, Suderman M, Blanchette M, Dostie J: ***HOX* in motion: tracking *HOXA* cluster conformation during differentiation.** *Nucleic Acids Res* 2013, Oct 29. [Epub ahead of print]

6. Fraser J, Rousseau M, Shenker S, Ferraiuolo MA, Hayashizaki Y, Blanchette M, Dostie J: **Chromatin conformation signatures of cellular differentiation.** *Genome Biol* 2009, **10:**R37.

7. Rousseau M, Fraser J, Ferraiuolo MA, Dostie J, Blanchette M: **Three-dimensional modeling of chromatin structure from interaction frequency data using Markov chain Monte Carlo sampling.** *BMC Bioinformatics* 2011, **12:**414.

8. Nakazawa SK, K,; Sasaki, Y. : **"Null" leukemic cell line (KOPN) - establishment and characterization.** *Jpn J Clin Hematol* 1978, **20**.

9. Lanotte M, Martin-Thouvenin V, Najman S, Balerini P, Valensi F, Berger R: **NB4, a maturation inducible cell line with t(15;17) marker isolated from a human acute promyelocytic leukemia (M3).** *Blood* 1991, **77:**1080-1086.

10. Hurwitz R, Hozier J, LeBien T, Minowada J, Gajl-Peczalska K, Kubonishi I, Kersey J: **Characterization of a leukemic cell line of the pre-B phenotype.** *Int J Cancer* 1979, **23:**174-180.

11. Schneider U, Schwenk HU, Bornkamm G: **Characterization of EBV-genome negative "null" and "T" cell lines derived from children with acute lymphoblastic leukemia and leukemic transformed non-Hodgkin lymphoma.** *Int J Cancer* 1977, **19:**621-626.

12. Andrews PW, Damjanov I, Simon D, Banting GS, Carlin C, Dracopoli NC, Fogh J: **Pluripotent embryonal carcinoma clones derived from the human teratocarcinoma cell line Tera-2. Differentiation in vivo and in vitro.** *Lab Invest* 1984, **50:**147-162.

13. Herrmann R, Han T, Barcos MP, Lok MS, Henderson ES: **Malignant lymphoma of pre-T-cell type terminating in acute myelocytic leukemia. A case report with enzymic and immunologic marker studies.** *Cancer* 1980, **46:**1383-1388.

14. Karpas A, Sandler RM, Thorburn RJ: **Null-cell properties of a lymphoid cell line from a child with acute lymphoblastic leukaemia.** *British journal of cancer* 1977, **36:**177-186.

15. Stong RC, Korsmeyer SJ, Parkin JL, Arthur DC, Kersey JH: **Human acute leukemia cell line with the t(4;11) chromosomal rearrangement exhibits B lineage and monocytic characteristics.** *Blood* 1985, **65:**21-31.

16. Kato YO, M.; Okumura, M.; Morishima, Y.; Horita, T.; Ohno, R.; Saito, H.; & Hirabayashi, N.: **Establishment of peroxidase positive, human monocytic leukemia cell line (NOMO-1) and its characteristics.** *Acta Haematol Jpn* 1986, **49**.

17. Collins SJ, Gallo RC, Gallagher RE: **Continuous growth and differentiation of human myeloid leukaemic cells in suspension culture.** *Nature* 1977, **270:**347-349.

18. Andrews PW: **Retinoic acid induces neuronal differentiation of a cloned human embryonal carcinoma cell line in vitro.** *Dev Biol* 1984, **103:**285-293.

19. Sundstrom C, Nilsson K: **Establishment and characterization of a human histiocytic lymphoma cell line (U-937).** *Int J Cancer* 1976, **17:**565-577.

20. Minowada J, Onuma T, Moore GE: **Rosette-forming human lymphoid cell lines. I. Establishment and evidence for origin of thymus-derived lymphocytes.** *J Natl Cancer Inst* 1972, **49:**891-895.

21. Ferraiuolo MA, Sanyal A, Naumova N, Dekker J, Dostie J: **From cells to chromatin: capturing snapshots of genome organization with 5C technology.** *Methods* 2012, **58:**255-267.

22. Miele A, Gheldof N, Tabuchi TM, Dostie J, Dekker J: **Mapping chromatin interactions by Chromosome Conformation Capture (3C).** In *Current Protocols in Molecular Biology.* *Volume* Supplement 74. Edited by Ausubel FM, R. Brent, R.E. Kingston, D.D. Moore, J.G. Seidman, J.A. Smith, and K. Struhl. Hoboken, N.J.: John Wiley & Sons; 2006: 21.11.21-21.11-20

23. <http://Dostielab.biochem.mcgill.ca> [<http://Dostielab.biochem.mcgill.ca>]

24. Nuwaysir EF, Huang W, Albert TJ, Singh J, Nuwaysir K, Pitas A, Richmond T, Gorski T, Berg JP, Ballin J, et al: **Gene expression analysis using oligonucleotide arrays produced by maskless photolithography.** *Genome Res* 2002, **12:**1749-1755.

25. Kim TH, Barrera LO, Zheng M, Qu C, Singer MA, Richmond TA, Wu Y, Green RD, Ren B: **A high-resolution map of active promoters in the human genome.** *Nature* 2005, **436:**876-880.

26. Selzer RR, Richmond TA, Pofahl NJ, Green RD, Eis PS, Nair P, Brothman AR, Stallings RL: **Analysis of chromosome breakpoints in neuroblastoma at sub-kilobase resolution using fine-tiling oligonucleotide array CGH.** *Genes Chromosomes Cancer* 2005, **44:**305-319.

27. Dostie J, Richmond TA, Arnaout RA, Selzer RR, Lee WL, Honan TA, Rubio ED, Krumm A, Lamb J, Nusbaum C, et al: **Chromosome Conformation Capture Carbon Copy (5C): a massively parallel solution for mapping interactions between genomic elements.** *Genome Research* 2006, **16:**1299-1309.

28. Dostie J, Dekker J: **Mapping networks of physical interactions between genomic elements using 5C technology.** *Nat Protoc* 2007, **2:**988-1002.
