# Supplementary material for: Classifying leukemia types with chromatin conformation data
Source: Genome Biol. 2014 Apr 30;15(4):R60. doi: 10.1186/gb-2014-15-4-r60 (PMC4038739; doi:10.1186/gb-2014-15-4-r60)
Supplement: Additional file 2: Table S1 — HOXA gene expression in leukemia cell samples used in this study. This table contains the mRNA quantification of all HOXA genes by RT-qPCR in cell lines used to compare classification by gene expression and chromatin conformation (Additional file 5: Figure S4). [file gb-2014-15-4-r60-S2.pdf]

Table S1. *HOXA* gene expression in leukemia cell samples used in this study.

Sample number	Sample name	*HOXA1*	*HOXA2*	*HOXA3*	*HOXA4*	*HOXA5*	*HOXA6*	*HOXA7*	*HOXA9*	*HOXA10*	*HOXA11*	*HOXA13*	MLL state	Leukemia Type
1	AF9-A-1	0.01*	0.05	0.06	0.06	0.04	0.01	0.68	3.04	3.55	0.04	0.00	AF9	AML
		0.00**	0.01	0.01	0.00	0.01	0.00	0.01	0.12	0.13	0.00	0.00		
2	AF9-A-2	0.01	0.05	0.08	0.04	0.00	0.00	0.63	2.26	3.01	0.04	0.00	AF9	AML
		0.00	0.01	0.00	0.00	0.00	0.00	0.03	0.15	0.19	0.00	0.00		
3	THP1-A-1	0.30	0.04	0.00	0.07	0.42	0.05	0.04	3.30	6.34	2.56	2.23	AF9	AML
		0.01	0.00	0.00	0.01	0.02	0.01	0.00	0.09	0.23	0.08	0.09		
4	THP1-B-1	0.28	0.07	0.01	0.02	0.09	0.01	0.02	2.29	4.50	1.33	0.31	AF9	AML
		0.00	0.00	0.00	0.00	0.02	0.00	0.00	0.13	0.17	0.14	0.01		
5	THP1-C-1	0.88	0.17	0.03	0.04	0.34	0.02	0.01	6.69	9.05	3.40	0.97	AF9	AML
		0.02	0.01	0.00	0.00	0.04	0.00	0.00	0.24	0.92	0.11	0.07		
6	THP1-D-1	1.44	2.49	0.26	0.15	0.53	0.04	0.01	2.81	3.61	1.61	0.65	AF9	AML
		0.05	0.13	0.00	0.00	0.01	0.00	0.00	0.16	0.25	0.06	0.05		
7	THP1-E-1	1.16	1.48	0.32	0.29	1.30	0.11	0.01	4.63	5.19	1.72	0.49	AF9	AML
		0.28	0.12	0.00	0.01	0.02	0.01	0.00	0.31	0.16	0.15	0.05		
8	THP1-E-2	-	-	0.19	0.05	0.88	-	-	2.05	6.84	2.06	1.67	AF9	AML
		-	-	0.02	0.00	0.02	-	-	0.06	0.66	0.12	0.09		
9	THP1-F-1	-	-	0.30	0.05	0.97	-	-	8.58	23.12	5.15	11.61	AF9	AML
		-	-	0.04	0.00	0.05	-	-	0.57	0.31	0.40	0.88		
10	ML-2	0.55	0.07	0.00	0.13	0.44	0.09	1.17	6.61	4.91	1.83	4.04	AF6	AML
		0.03	0.00	0.00	0.01	0.02	0.00	0.04	0.28	0.11	0.09	0.17		
11	NOMO-1	0.29	-	0.05	0.56	1.70	0.34	3.63	5.93	7.44	2.12	0.63	AF9	AML
		0.01	-	0.01	0.04	0.06	0.02	0.03	0.28	0.48	0.08	0.09		
12	ENL-A-1	0.00	0.00	0.00	0.00	0.00	0.00	0.19	0.84	0.37	0.00	0.00	ENL	ALL
		0.00	0.00	0.00	0.00	0.00	0.00	0.01	0.03	0.02	0.00	0.00		
13	ENL-A-2	0.01	0.03	0.11	0.15	0.13	0.02	0.08	0.20	0.23	0.00	0.00	ENL	ALL
		0.00	0.00	0.00	0.01	0.00	0.00	0.01	0.03	0.05	0.00	0.00		
14	ENL-A-3	0.01	0.03	0.06	0.08	0.00	0.01	0.03	0.11	0.07	0.00	0.00	ENL	ALL
		0.00	0.00	0.00	0.01	0.00	0.00	0.00	0.01	0.01	0.00	0.00		
15	ENL-B-2	0.00	0.05	0.00	0.01	0.00	0.00	0.17	0.72	0.52	0.00	0.00	ENL	ALL
		0.00	0.00	0.00	0.00	0.00	0.00	0.01	0.05	0.03	0.00	0.00		
16	ENL-B-4	0.00	0.09	-	-	-	-	1.54	3.38	2.29	0.00	0.00	ENL	ALL
		0.00	0.01	-	-	-	-	0.08	0.17	0.07	0.00	0.00		
17	KOPN8-1	0.38	0.15	0.10	0.47	7.04	1.08	4.71	5.38	21.18	3.70	0.04	ENL	ALL
		0.01	0.01	0.00	0.02	0.12	0.03	0.11	0.09	0.52	0.13	0.01		
18	RS4;11	0.27	0.03	0.02	0.29	1.26	0.49	8.10	6.18	12.26	0.26	0.02	AF4	ALL
		0.01	0.00	0.00	0.02	0.19	0.01	0.59	0.35	0.85	0.01	0.00		
19	Karpas-45	0.00	0.00	0.00	0.00	0.00	0.00	0.00	0.00	0.00	0.00	0.01	AFX	ALL
		0.00	0.00	0.00	0.00	0.00	0.00	0.00	0.00	0.00	0.00	0.00		
20	NB4-1	0.40	0.03	-	-	-	-	0.00	0.00	1.88	0.10	-	wt	AML
		0.02	0.00	-	-	-	-	0.00	0.00	0.15	0.00	-		
21	NB4-3	1.18	0.05	0.01	0.31	0.08	0.01	0.03	0.09	8.65	2.58	1.81	wt	AML
		0.01	0.01	0.00	0.02	0.01	0.00	0.00	0.00	0.23	0.06	0.04		
22	HL60	0.78	0.04	0.00	0.27	1.49	0.07	2.05	5.93	7.42	5.33	2.16	wt	AML
		0.02	0.00	0.00	0.01	0.05	0.00	0.06	0.21	0.37	0.16	0.09		
23	NALM6-4	0.00	0.00	0.00	0.00	0.00	0.00	0.00	0.00	0.00	0.00	0.00	wt	ALL
		0.00	0.00	0.00	0.00	0.00	0.00	0.00	0.00	0.00	0.00	0.00		
24	NALM6-5	0.00	0.00	0.00	0.00	0.00	0.00	0.00	0.00	0.00	0.00	0.00	wt	ALL
		0.00	0.00	0.00	0.00	0.00	0.00	0.00	0.00	0.00	0.00	0.00		
25	Jurkat	0.55	0.07	0.01	0.10	1.72	0.20	1.85	0.01	1.30	0.04	0.08	wt	ALL
		0.02	0.01	0.00	0.01	0.06	0.01	0.06	0.00	0.09	0.00	0.01		
26	U937	0.37	0.05	0.01	0.10	0.69	0.13	0.28	6.64	19.61	5.60	8.72	wt	ALL
		0.01	0.00	0.00	0.01	0.05	0.01	0.01	0.16	0.26	0.15	0.33		
27	MOLT-4	0.23	0.02	0.00	0.00	0.00	0.00	0.01	0.00	0.01	0.00	0.00	wt	ALL
		0.03	0.00	0.00	0.00	0.00	0.00	0.00	0.00	0.00	0.00	0.00		
28	NT2D1-2	0.01	0.00	0.00	0.00	0.01	0.00	0.05	0.00	0.00	0.00	0.00	wt	EC
		0.00	0.00	0.00	0.00	0.00	0.00	0.00	0.00	0.00	0.00	0.00		
29	NT2D1-3	0.03	0.00	0.00	0.00	0.01	0.00	0.06	0.00	0.00	0.00	0.00	wt	EC
		0.00	0.00	0.00	0.00	0.00	0.00	0.00	0.00	0.00	0.00	0.00		
30	NT2D1-24h	80.60	3.91	0.08	0.06	0.71	0.01	0.33	0.02	-	0.03	0.03	wt	EC
		1.63	0.07	0.00	0.00	0.01	0.00	0.01	0.00	-	0.00	0.00		

*Each entry corresponds to mRNA expression levels relative to actin ($X10^{-3}$)
** values under each entry represents the standard error of the mean (s.e.m.)
