# Supplementary figures and images for: Classifying leukemia types with chromatin conformation data
Source: Genome Biol. 2014 Apr 30;15(4):R60. doi: 10.1186/gb-2014-15-4-r60 (PMC4038739; doi:10.1186/gb-2014-15-4-r60)

**A**

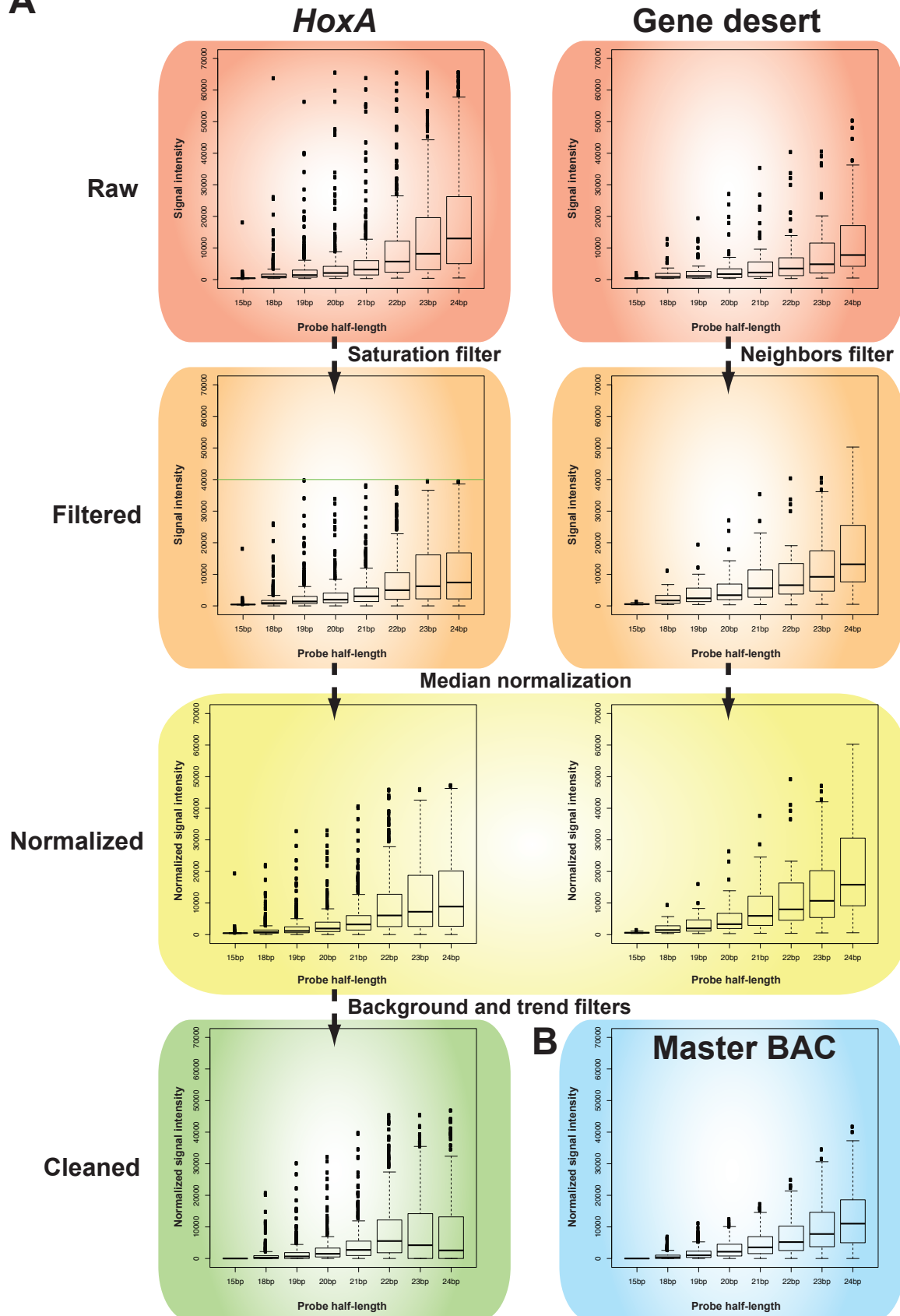

Supplement: Additional file 3: Figure S1 — Workflow of 5C data processing, normalization, and conversion into interaction frequencies. This figure illustrates our approach to calculate normalized IFs from 5C data. This method and Additional file 3: Figure S1 are described in detail in (Additional file 1: Supplementary Materials and methods and Additional file 3: Figure S1). [file gb-2014-15-4-r60-S3.pdf]

**A**

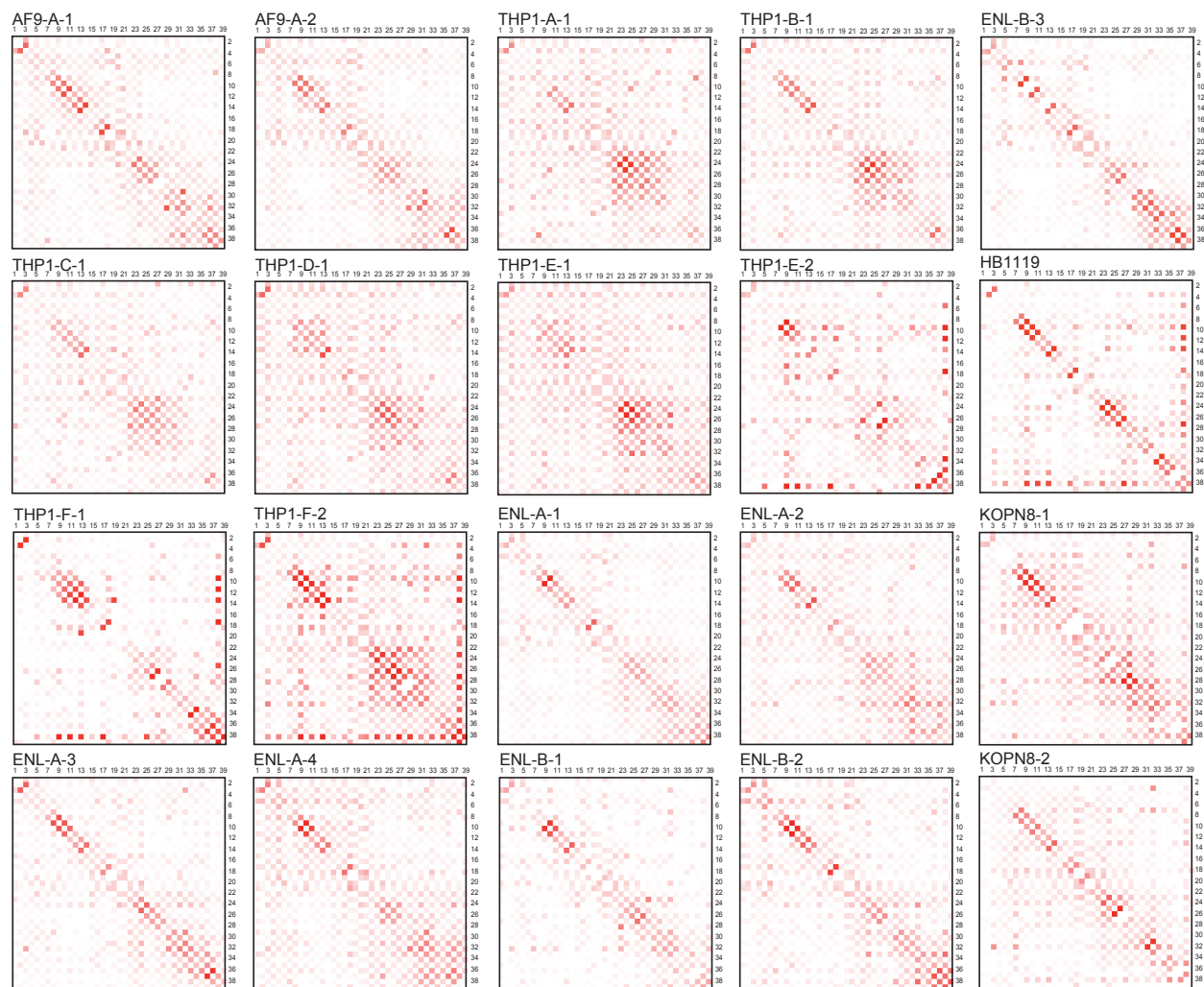

**B**

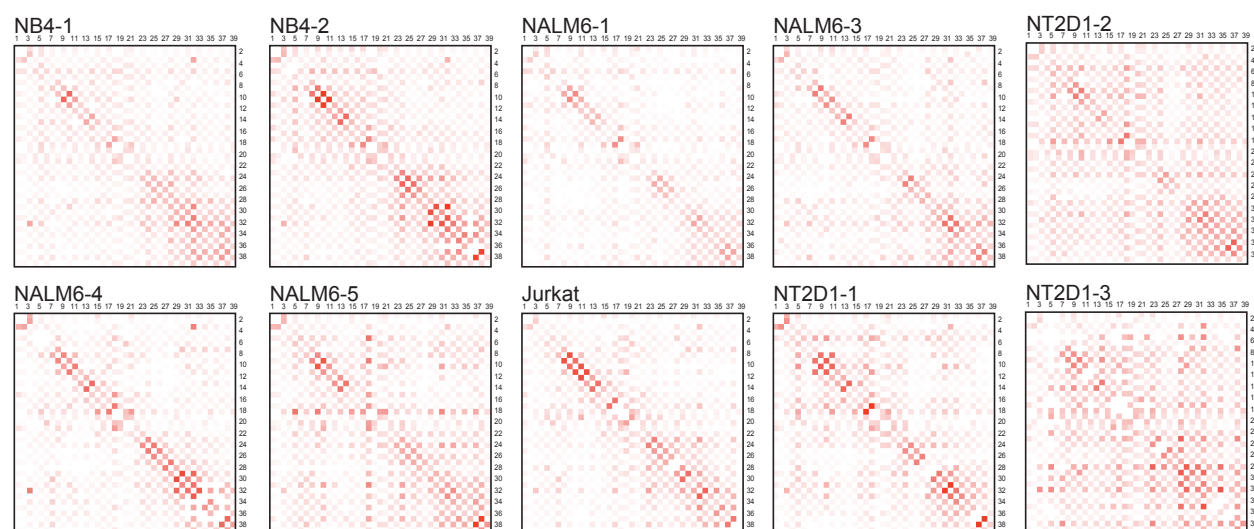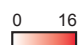

Supplement: Additional file 4: Figure S2 — 5C datasets generated for the 3D-SP training set. This figure shows the 5C data of the cell samples from the training set in the form of heatmaps as described in Additional file 4: Figure S2 (Additional file 1). [file gb-2014-15-4-r60-S4.pdf]

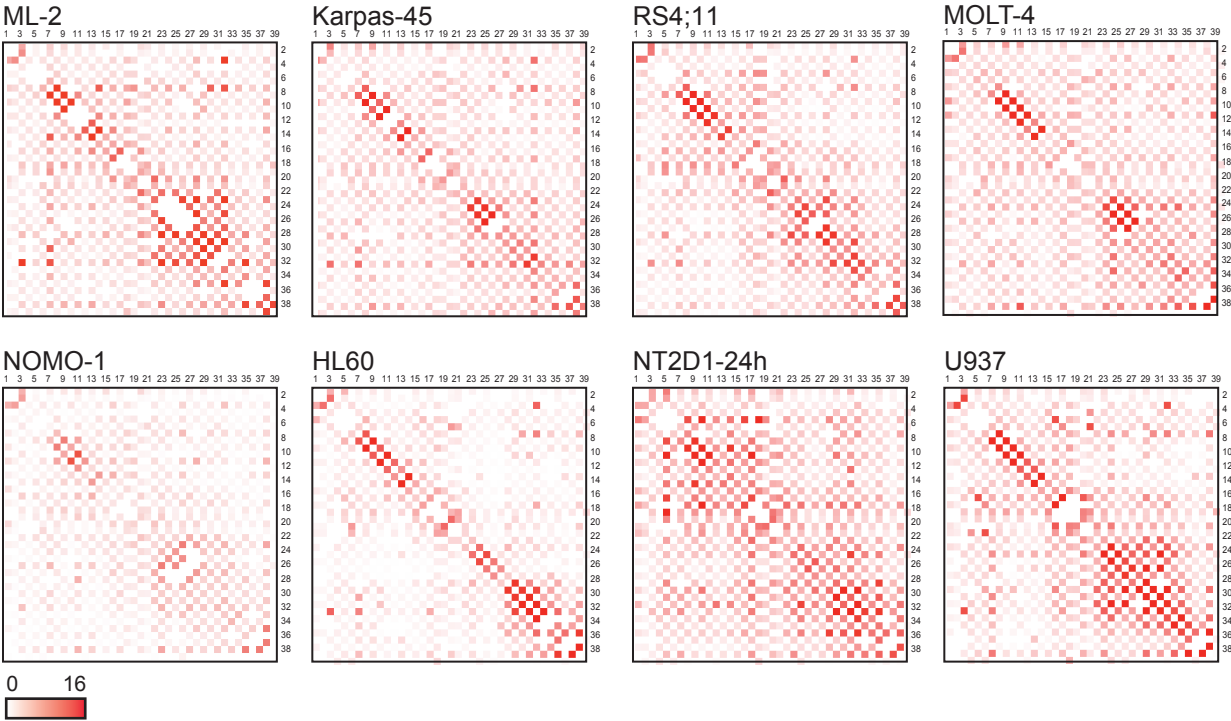

Supplement: Additional file 6: Figure S3 — 5C datasets generated for the 3D-SP test set. This figure shows the 5C data of the cell samples from the test set in the form of heatmaps as described in Additional file 6: Figure S3 (Additional file 1). [file gb-2014-15-4-r60-S6.pdf]
