# Supplementary material for: Classifying leukemia types with chromatin conformation data
Source: Genome Biol. 2014 Apr 30;15(4):R60. doi: 10.1186/gb-2014-15-4-r60 (PMC4038739; doi:10.1186/gb-2014-15-4-r60)
Supplement: Additional file 5: Figure S4 — 3D-SP performs better than gene expression to classify MLL leukemia types. This figure compares classification of leukemia cell samples using HOXA gene expression and HOXA chromatin organization. The figure is described in the Additional file 5: Figure S4 in Additional file 1. [file gb-2014-15-4-r60-S5.pdf]

A

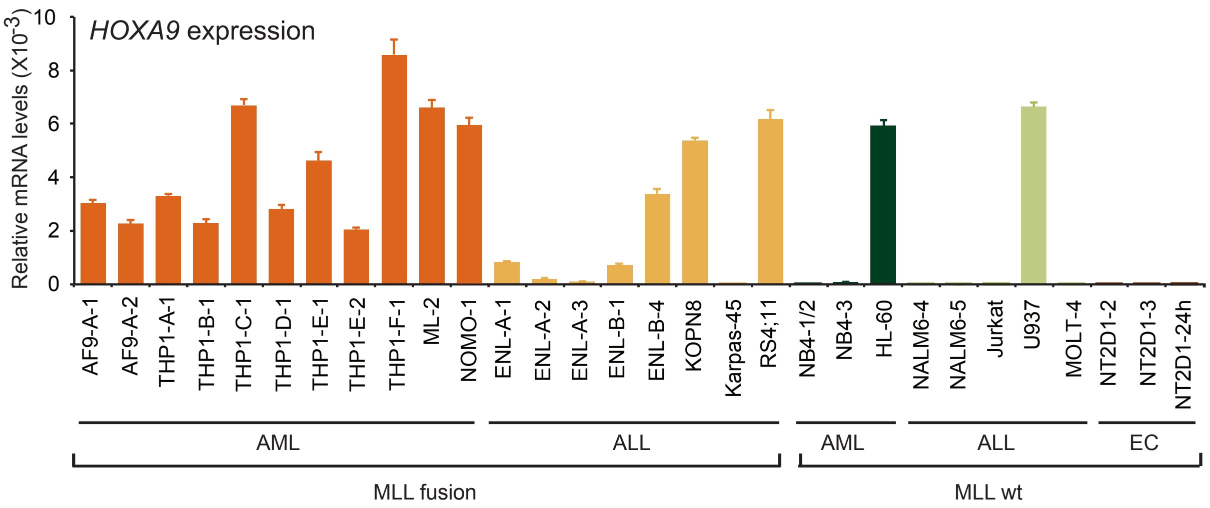

B

Sample number	Sample name	MLL state	Leukemia type
1	AF9-A-1	AF9	AML
2	AF9-A-2	AF9	AML
3	THP1-A-1	AF9	AML
4	THP1-B-1	AF9	AML
5	THP1-C-1	AF9	AML
6	THP1-D-1	AF9	AML
7	THP1-E-1	AF9	AML
8	THP1-E-2	AF9	AML
9	THP1-F-1	AF9	AML
10	ML-2	AF6	AML
11	NOMO-1	AF9	AML
12	ENL-A-1	ENL	ALL
13	ENL-A-2	ENL	ALL
14	ENL-A-3	ENL	ALL
15	ENL-B-2	ENL	ALL
16	ENL-B-4	ENL	ALL
17	KOPN8-1	ENL	ALL
18	Karpas-45	AFX	ALL
19	RS4;11	AF4	ALL
20	NB4-1	wt	AML
21	NB4-3	wt	AML
22	HL60	wt	AML
23	NALM6-4	wt	ALL
24	NALM6-5	wt	ALL
25	Jurkat	wt	ALL
26	U937	wt	ALL
27	MOLT-4	wt	ALL
28	NT2D1-2	wt	EC
29	NT2D1-3	wt	EC
30	NT2D1-24h	wt	EC

C

Classification of MLL fusion leukemia type

Input	*Decision tree (DT)*	*Support vector machine*
HOXA9 gene expression	86%	48%
All *HOXA* gene expression	83%	62%
5C data	76%	93%

D

***HOXA9* DT classification**

Actual state	*Predicted fusion*	*Predicted wt*
MLL fusion	18	1
MLL wt	3	7

3D-SP classification

Actual state	*Predicted fusion*	*Predicted wt*
MLL fusion	19	0
MLL wt	2*	8

*Improperly classified samples were also incorrectly predicted by the *HOXA9* DT approach.
